# Secure Service Proxy: A CoAP(s) Intermediary for a Securer and Smarter Web of Things

**DOI:** 10.3390/s17071609

**Published:** 2017-07-11

**Authors:** Floris Van den Abeele, Ingrid Moerman, Piet Demeester, Jeroen Hoebeke

**Affiliations:** Ghent University - imec, IDLab, Department of Information Technology, Technologiepark Zwijnaarde 15, B-9052 Ghent, Belgium; ingrid.moerman@ugent.be (I.M.); piet.demeester@ugent.be (P.D.); jeroen.hoebeke@ugent.be (J.H.)

**Keywords:** CoAP, DTLS, REST, IoT, WoT, proxy, 6LoWPAN, CoRE, LLN

## Abstract

As the IoT continues to grow over the coming years, resource-constrained devices and networks will see an increase in traffic as everything is connected in an open Web of Things. The performance- and function-enhancing features are difficult to provide in resource-constrained environments, but will gain importance if the WoT is to be scaled up successfully. For example, scalable open standards-based authentication and authorization will be important to manage access to the limited resources of constrained devices and networks. Additionally, features such as caching and virtualization may help further reduce the load on these constrained systems. This work presents the Secure Service Proxy (SSP): a constrained-network edge proxy with the goal of improving the performance and functionality of constrained RESTful environments. Our evaluations show that the proposed design reaches its goal by reducing the load on constrained devices while implementing a wide range of features as different adapters. Specifically, the results show that the SSP leads to significant savings in processing, network traffic, network delay and packet loss rates for constrained devices. As a result, the SSP helps to guarantee the proper operation of constrained networks as these networks form an ever-expanding Web of Things.

## 1. Introduction

In recent years, the Internet of Things (IoT) has increasingly become a hot topic in industry, academia, the do-it-yourself community and also consumers. Businesses are attracted by the new product opportunities and new sources of revenue that the IoT promises to bring. For example, a 2013 market report on IoT by Cisco Inc. (San Jose, CA, USA) predicts 14.4 trillion USD in created value for the “Internet of Everything” from 2013 to 2022 [1]. Academia is interested in the many new problems and issues that arise when deploying billions of devices on the Internet. These issues include big data analytics, energy efficient communications, large-scale deployments, management of devices, communication protocols, security models, data privacy and many more. An introduction to the research aspect of the IoT is presented in [2]. Finally, consumers are drawn to the IoT because IoT products promise to bring improvements and novel services to their daily lives. Examples of IoT domains include smart home, smart health, smart transportation, smart factory, smart grid and many more [3].

As the Internet of Things continues to grow in scope and in size, the number of available technologies and platforms that promise to enable the IoT keeps increasing. As a family of such technologies, a complete protocol stack was standardized at the Internet Engineering Task Force (IETF) for use with constrained IoT devices in Low-power and Lossy Networks (LLNs) [4]. This suite of protocols defines the communication stack from the network layer up to the application layer. In contrast to the popular alternative ZigBee [5], the IETF protocol stack gives the developer more flexibility to model the network and the application to a specific use-case. For instance, with the IPv6 Routing Protocol for Low-power and Lossy Networks (RPL) [6] the routing can be tuned by employing different objective functions that optimize routes according to the metrics that are relevant to the use case (e.g., minimize hop count, maximize battery lifetime, etc.). Another example of flexibility is found at the application layer, where the REST architecture followed by the CoAP protocol allows developers to design their own RESTful resources and to model their behavior. In terms of security, the IETF elected to standardize an End-to-End (E2E) architecture as it is a popular choice on the unconstrained Web today. Therefore, the CoAP standard defines DTLS (i.e., Datagram TLS) as its recommended security method.

Secure Sockets Layer (SSL) and, later, Transport Layer Security (TLS) have been around since the end of the past century and have become very popular protocols for their roles in securing the WWW. Today, (D)TLS has become a flexible protocol where endpoints can negotiate the type of security and where a built-in extension mechanism allows one to add new features to the protocol without touching the base specification. A comprehensive overview of the (D)TLS protocol is presented in the Background Section 2. Widespread adoption, a wide range of implementations, an open protocol specification and a high level of interoperability are just a few of the benefits of the TLS protocol. Nevertheless, one should be careful when deploying end-to-end security with DTLS in constrained environments. This issue has been recognized by the IETF, which has formulated guidance for implementing and deploying DTLS in constrained environments in Request for Comments (RFC) 7925 [7].

Despite the advantages offered by DTLS, E2E security has a number of disadvantages when deployed as-is in LLNs. One issue with E2E security is that it completely blocks out any third party (e.g., intermediate middleboxes) from taking part in the communication. In most traditional Internet deployments, this is a wanted property of E2E security, but in LLNs, it stops intermediary systems from providing services that can improve resource usage and the performance of constrained devices and networks. For example, caching of CoAP responses is not possible when E2E security is applied between the CoAP client and the constrained CoAP server. A second disadvantage of E2E security is that application-layer enhancements cannot be applied by middleboxes, as all communication is enciphered. Thus, access control, admittance control and other similar features cannot be provided at the edge of the LLN. Another known problem with DTLS is its performance in duty-cycled networks, which is common in multi-hop LLNs. Research [8] has shown that the latency introduced by the DTLS handshake can become excessively large in multi-hop duty-cycled networks (up to 50 s for four hops). Vučinć et al. also show that constrained nodes can only store a limited number of DTLS sessions in their memory (e.g., max. three DTLS sessions for a WiSMote node). As a result, nodes have to start dropping active DTLS sessions from memory, which can deteriorate battery lifetime and DTLS performance. Finally, end-to-end network addressing reduces the effectiveness of 6LoWPAN compression. This is due to the fact that the IPv6 prefixes for nodes situated on the Internet and the used UDP ports are difficult or impossible to compress on 6LoWPAN. All of these issues are covered in greater depth in the problem statement, cf. Section 3.

The goal of this work is to overcome the issues identified with E2E security without losing the benefits offered by such a widely-used protocol as DTLS. To this end, we propose the “Secure Service Proxy” (SSP). It is a reverse DTLS and CoAP proxy that provides a secure bridge between clients on the Internet and constrained IoT devices in a low-power and lossy network. By employing DTLS on both legs of the communication path, the resulting system can still enjoy most of the benefits offered by the popularity of DTLS without suffering from the disadvantages of E2E security specific to constrained environments (as identified in the previous paragraph). As the SSP operates as a trusted entity in the network, it can also offer network services such as caching, as well as application-layer enhancements. For the latter, this paper employs the concept of node virtualization where a constrained node has a virtual counterpart that resides on the proxy and that offers additional functionality on behalf of the node. This virtualization concept is effective because the SSP is deployed on hardware more powerful than the constrained nodes themselves. As a result, node virtualization can offer new and complex functionality that is unfeasible to offer on the constrained node itself. Examples include support for more complex modes of DTLS (e.g., public key infrastructure and certificate-based suites), translating responses between content formats, offering verbose semantic descriptions for the constrained node, storing large binary blobs (e.g., a picture of the deployment area), keeping historical data, etc.

Our contributions in this paper are as follows. First, we identify and discuss a number of issues with end-to-end security in constrained RESTful environments. We argue that these issues can be overcome by a reverse proxy approach that splits the end-to-end security at the proxy. Secondly, we design and implement such a reverse proxy. Apart from solving the E2E security issues, our developed proxy can also offer additional functionality and services on behalf of the constrained network and the constrained nodes. To our knowledge, this work is the first to study, design, implement and evaluate a reverse proxy for use with end-to-end security in constrained RESTful environments. Finally, by means of a real-world evaluation, we show that our work can significantly improve the operation of constrained networks by reducing power consumption, network latency and network traffic.

The rest of this paper is structured as follows. First, a brief overview of CoAP and DTLS is presented in the next section. Using this overview, a number of issues with deploying CoAP and DTLS in low-power and lossy networks is presented in Section 3. This section also lists the research goals of this work. In Section 4, our approach to tackling these issues is presented together with the design of the secure service proxy and an overview of the security risks related to breaking end-to-end security. The secure service proxy is aligned to similar work in the literature and the commercial world in Section 5. An extensive evaluation of our approach based on both simulations and a real-world wireless sensor network testbed is presented in Section 6. Section 7 presents the conclusions that are drawn from this work.

## 2. Overview of CoAP and DTLS

### 2.1. The Constrained Application Protocol

RFC 7252 [9] states that the Constrained Application Protocol (CoAP) is a specialized Web transfer protocol for use with constrained nodes and constrained networks in the Internet of Things. The protocol is designed for Machine-to-Machine (M2M) applications such as smart energy and building automation. The main design considerations for CoAP include simplicity, very low overhead, easy translation to and from HTTP and support for multicast.

In CoAP, constrained devices that host applications structure their data and actions as RESTful Web services, also called CoAP resources. CoAP clients send requests to resources in order to retrieve and store data or trigger actions. CoAP defines the same request methods as HTTP: GET, PUT, POST and DELETE. They are used respectively for retrieving data, storing data, toggling an action and removing data. CoAP chose UDP as its transport protocol due to the lightweight nature of UDP (TCP was deemed too verbose due to its connections and too complex to implement in constrained devices). Therefore, CoAP includes a simple reliability layer and deduplication mechanism in order to compensate for the minimalistic nature of UDP. In order to minimize overhead, CoAP uses a binary format for encoding message options in the headers of CoAP requests and responses. As a result, the CoAP message size is significantly reduced when compared to a non-binary encoded protocol, such as HTTP [10], which is important in LLNs where message sizes are typically small and communication is expensive for battery-powered devices.

**Figure 1:**
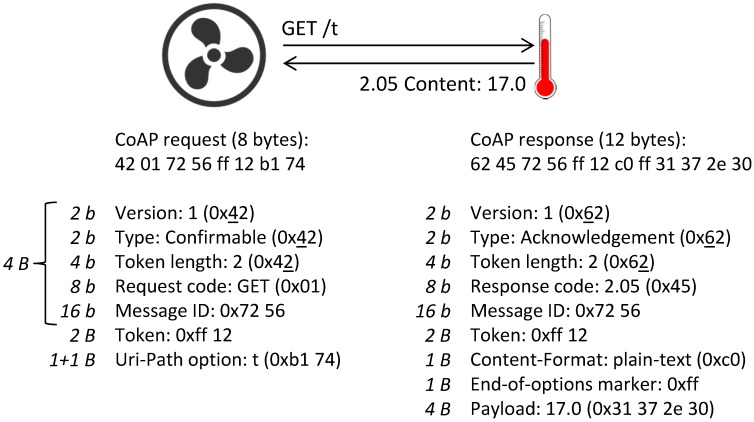
Anatomy of a typical CoAP request and response.

An illustration of a typical CoAP request/response exchange is shown in Figure 1, where a client (a ventilation unit) retrieves a temperature resource on a CoAP server. The first elements of the CoAP header are the two-bit protocol version (RFC 7252 standardizes Version 1) and the two-bit message type. By sending a confirmable message, a sender can ask a receiver to acknowledge the reception of a message. This is reflected in the message type of the response, which is an acknowledgment. In most cases (like here), the response message is actually piggy-backed on the acknowledgment message in order to reduce the number of messages. The four-bit token length comes after the message type in the CoAP header, and it represents the length of the optional message token in bytes. The next element of the CoAP header is the eight-bit message code, which consists of a three-bit class and a five-bit subfield. Requests codes are Class 0 codes (e.g., GET is code 0.01), and successful response codes are Class 2 codes (e.g., Content is code 2.05). The final part of the fixed four-byte CoAP header is the two-byte message ID. It is used for deduplication and for confirmable (CON) messages, where acknowledgments echo the message ID of the CON message.

The token is used to match a response with a request and can vary in length between zero and eight bytes. After the token come the header options and the payload (if any). In CoAP, header options are assigned unique numbers by the Internet Assigned Numbers Authority (IANA) and are delta encoded in CoAP messages in order to reduce their encoding size. Every option encoding contains the delta of the option number (relative to the preceding option), the size of the value of the option (in bytes) and the value of the option. Finally, the options and the payload are separated by an end-of-options marker (0xff).

The CoAP Observe option [11] is a CoAP protocol extension that is important for this work. When a client is observing a REST resource on a CoAP server, the server will notify the client of state changes for that resource. This frees the client from polling the resource on the server, which can save resources in LLNs when changes in resource state occur rarely. RFC 7641 [11] also states that intermediaries must aggregate observation registrations: “If two or more clients have registered their interest in a resource with an intermediary, the intermediary MUST register itself only once with the next hop and fan out the notifications it receives to all registered clients. This relieves the next hop from sending the same notifications multiple times and thus enables scalability”. Apart from enabling scalability, aggregation also saves resources.

### 2.2. Datagram Transport Layer Security

For security, CoAP standardized end-to-end security and DTLS as its default security mechanism and protocol respectively. The primary motivation for preferring transport-layer security over alternatives such as object security and network layer security is the popularity of TLS on the conventional Web. Datagram TLS is by design very similar to the TLS protocol, and the specification of DTLS is largely written as a set of changes to the TLS specification [12]. However, there are some key differences as DTLS runs over an unreliable datagram transport while TLS runs over a reliable TCP transport. Therefore, DTLS must cope with the reliable and ordered delivery of packets as available in TLS. To this end, DTLS introduces a simple timeout and retransmission scheme and adds an explicit sequence number to the Record Protocol (versus an implicit number as available via TCP in TLS). Another difference is that stream ciphers must not be used with DTLS. DTLS also enhanced the handshake protocol with a stateless cookie exchange for denial of service resistance. By forcing DTLS clients to echo the cookie in their second handshake message, malicious clients (e.g., those spoofing IP addresses) can be rooted out, and a DTLS server can avoid wasting resources on bogus handshakes.

DTLS is a session-based protocol in that DTLS endpoints have to set up a session when they want to communicate securely. Negotiation of the security parameters for the session and peer authentication are both performed during the handshake phase of the protocol. After the handshake phase, both endpoints can exchange data with guarantees for confidentiality, endpoint authentication and integrity of the data. To this end, DTLS employs symmetric cryptography for data encryption according to an encryption algorithm and encryption keys that are agreed upon during the handshake. DTLS also guarantees message integrity by means of Hash-based Message Authentication Codes (HMAC). Sessions are typically negotiated on an ad hoc basis, although long-term sessions and resumption of established sessions are possible in DTLS.

TLS introduces the concept of cipher suites; these are named combinations of the authentication and key exchange algorithm, the cipher and key length, the cipher mode of operation, the hash algorithm for integrity protection and the hash algorithm for use with pseudorandom functions.

**Figure 2:**
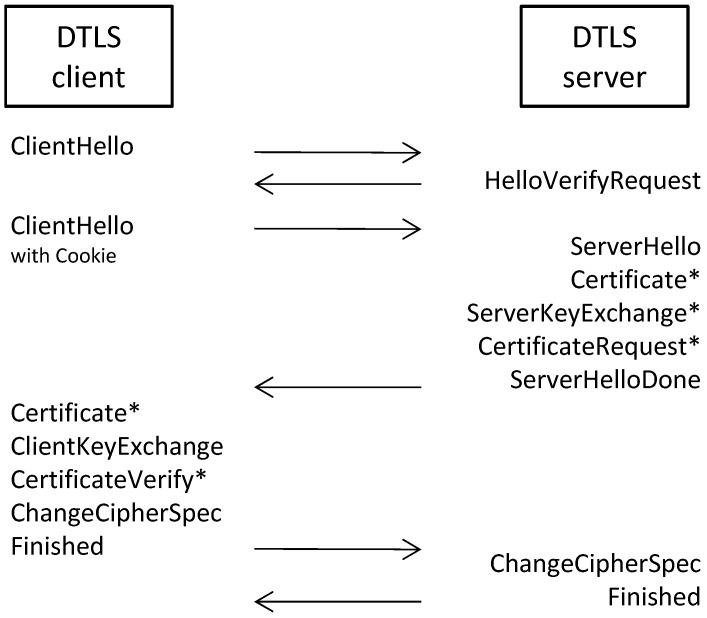
The full DTLS handshake.

The DTLS handshake is shown in Figure 2. In order to reduce the number of network packets, multiple DTLS messages can be grouped into a single flight of messages. In the figure, the horizontal arrows correspond to the different message flights. The DTLS client initiates the handshake with the ClientHello message, to which the server replies with a HelloVerifyRequest message. The HelloVerifyRequest message contains the stateless cookie for DoS mitigation and must be echoed by the client in its second ClientHello message. After the server has verified the cookie, it responds with the ServerHello message. The hello messages are used to establish security enhancement capabilities between the client and server [13]. They establish the following attributes: protocol version, session ID (used in session resumption), cipher suite and compression method. Additionally, two random values are generated and exchanged: one for the client and one for the server.

The messages of the remainder of the handshake depend on the negotiated security enhancement capabilities. In the figure, messages marked with an asterisk (*) are optional or situation-dependent messages. The figure shows the message flow for a certificate-based cipher suite where the server replies with Certificate, ServerKeyExchange, CertificateRequest and ServerHelloDone messages. If the cipher suite requires the server to authenticate itself, then the server sends its X.509 certificate in a Certificate message. In cases where the key exchange does not use the server certificate, the server may send a ServerKeyExchange message. For example, in Pre-Shared Key cipher suites (PSK suites are discussed later), the server may send a hint in the ServerKeyExchange message to help the client in selecting which PSK identity to use. Additionally, the server may also send a CertificateRequest message to request a certificate from the client. Finally, a ServerHelloDone message is sent by the server to indicate that the hello-message phase of the handshake is complete.

If the server requested a certificate, the client must provide one in its Certificate message. Next, the client sends a ClientKeyExchange message, the contents of which depend on the chosen key exchange algorithm. In the case of RSA for example, the client chooses a secret and encrypts it with the public key from the certificate of the server and sends the result in the ClientKeyExchange message. Together with the Certificate and ServerKeyExchange messages of the server, the client’s Certificate and ClientKeyExchange messages are used for the key exchange. The CertificateVerify message allows the client to prove the possession of the private key in the certificate. In the case of pre-shared key cipher suites, the key exchange of the client consists of a ClientKeyExchange message, which contains the identity of the chosen PSK.

Next, the client sends a ChangeCipherSpec message, which signals that the client has switched to the negotiated cipher spec. The client then immediately sends the Finished message, which contains a hash of the shared secret and all handshake messages. The server must verify the contents of the Finished message in order to detect any tampering of the handshake messages. The Finished message also proves that the client knows the correct shared secret (i.e., the pre-master secret), and any subsequent keying material (master secret, encryption keys and MAC keys) is generated from this pre-master secret. After the server has sent its own ChangeCipherSpec and Finished messages and the client has successfully verified the Finished message, the handshake is completed, and secure communication of application data can start.

### 2.3. DTLS in Constrained Environments

There are a number of additional protocol features that are applicable to DTLS in constrained environments, and these are discussed in this subsection. RFC 5116 [14] introduced Authenticated Encryption with Associated Data (AEAD) to TLS, which enables the use of cipher suites that use the same cipher for confidentiality, authenticity and integrity protection. Particularly in constrained environments, AEAD provides the benefit of more compact implementations as only one cipher has to be implemented.

RFC 6655 [15] defines multiple such compact cipher suites that use the widespread AES cipher in the Counter with Cipher Block Chaining-Message Authentication Code (CBC-MAC) Mode (CCM). AES is a popular choice in constrained environments, as it is often accelerated in hardware in modern IoT systems (e.g., the TI CC2538 SoC has an AES accelerator on the same die as the ARM-M3 CPU). Note that the AEAD construct is only supported from Version 1.2 of the DTLS protocol.

RFC 4279 [16] introduces the Pre-Shared Key (PSK) cipher suites for TLS. These cipher suites are interesting for constrained devices, as the size of the key exchange is minimal: typically only a PSK identifier in the client key exchange is exchanged. Of course, key management is an important issue in this case, as common cryptography practice dictates that a unique PSK should be allocated for every peer. The ‘TLS_PSK_WITH_AES_128_CCM_8’ cipher suite combines the benefits of PSKs and AES-CCM in that only one cipher is needed (AES), and the key exchange is minimal. This cipher suite is also the mandatory-to-implement PSK cipher suite for DTLS in the CoAP RFC [9]. Furthermore, this suite uses just an eight-byte authentication tag (as opposed to a 16-byte tag), which is more suitable in networks where bandwidth is constrained and messages sizes may be small.

RFC 7250 [17] introduces a new certificate type and two TLS extensions for exchanging Raw Public Keys (RPKs) in DTLS. In this case, a peer has an asymmetric key pair, but it does not have an X.509 certificate; this asymmetric key pair is the RPK. This extension allows the raw public key to be used for authentication, which is beneficial in constrained environments as RPKs are smaller in size than X.509 certificates. Additionally the resulting key exchange is therefore smaller, as well. Of course, the scalability benefits of a Public Key Infrastructure (PKI) are lost when using RPKs.

Finally, RFC 7251 [18] describes the use of AES-CMM elliptic curve cryptography (ECC) cipher suites in DTLS. This type of cipher suites uses the AEAD mechanism to provide confidentiality, authenticity and integrity of application data with just AES, while using Ephemeral Elliptic Curve Diffie–Hellman (ECDHE) as their key exchange and peer authentication mechanisms. ECC is attractive for constrained environments as its smaller key sizes result in savings for power, memory, bandwidth and computational cost [19]. For example, a 256 to 383-bit ECC key is considered comparable in strength to a 3072-bit RSA key by NIST [20]. CoAP mandates the use of the ‘TLS_ECDHE_ECDSA_WITH_AES_128_CCM_8’ cipher suite for X.509 certificates in constrained environments. This cipher suite uses the secp256r1 or NIST P-256 elliptic curve.

## 3. Problem Statement and Research Goals

When securing communications in LLNs via end-to-end security with DTLS, one should be mindful of a number of potential issues and pitfalls. Some of these issues arise due to the limitations of the constrained devices that secure the communications. For example, in end-to-end security, there is a considerable difference between constrained devices (and their protocols) and powerful Internet hosts (and their protocols) in terms of available resources and design. A second potential issue stems from the DTLS protocol itself, namely the large overhead of the DTLS handshake can be an issue of concern in constrained networks. A third group of issues is related to securing the LLN itself and is the result of deploying end-to-end security in LLNs. Apart from these issues related to end-to-end security in LLNs, there is also the problem of the limited amount of application layer functionality that can be provided by constrained IoT devices. In a world as heterogeneous as the IoT there exists a need for protocol translation, data format mapping, semantic descriptions and many other features that improve the interoperability with IoT devices. Similarly, network access to constrained nodes and LLNs should be as efficient as possible by supporting caching of information, efficient discovery and network edge filtering. These types of functionality are too complex and in some cases impossible for implementation on a constrained device. Clearly, an approach that does not burden the constrained device is needed in this case. The remainder of this section discusses these various issues and problems in more detail.

### 3.1. End-To-End Security in LLNs

Constrained devices with a limited power source (e.g., battery powered or energy scavenging devices) should take care to avoid excessive network communications in order not to preemptively deplete the power source. Similarly, constrained networks where the available throughput is in the order of a few kbps should minimize the amount of network communications to avoid congestion. Therefore, chatty or verbose security protocols that communicate excessive amounts of information should be avoided in these situations. As DTLS employs UDP instead of TCP as its transport protocol, it avoids the TCP handshake, which reduces the number of messages exchanged between DTLS clients and servers. However, some options supported by DTLS, as presented in the previous section, may lead to large amounts of network communications. Specifically, certificate-based cipher suites involve sending the certificate of the DTLS server (and peer, depending on the security needs) over the network. These certificates are generally large (i.e., a thousand bytes or more), and therefore, their network communication can be problematic when communication has a large impact on the power source or the network. As a result, these types of devices are unable to offer authentication based on PKI certificates. While raw public keys are significantly more compact than X.509 certificates, they do not offer the same benefits in terms of authentication and scalability.

For devices with limited computational power (e.g., low-cost embedded systems) certain cryptographic primitives may prove too complex for computation by the low cost microcontroller. While hardware acceleration may help to alleviate this issue, it can be an expensive option and might only be available for certain primitives: e.g., AES is often accelerated in hardware, while others are not. Specifically, public-key cryptography methods (e.g., based on large integer factorization or discrete logarithm problems) and key agreement schemes (such as (EC)DH) may be too taxing for constrained microcontrollers. Therefore, the set of cryptographic functions that can be offered by such low cost embedded systems excludes a number of common cryptographic primitives and is typically limited to what can be achieved by symmetric-key cryptography.

Another important limitation in constrained environments is the low amount of available memory (i.e., both volatile and non-volatile memory). For example, according to IETF RFC 7228 [4], Class 1 constrained devices have around 10 kibibyte (KiB) of RAM and 100 KiB of ROM memory. Such a small amount of memory must accommodate an entire networking stack, adequate security mechanisms, peripheral control, the application itself and various other subsystems. This forces a device manufacturer to limit the amount of software that will ship with the device by carefully selecting what is needed. One consequence is that it is impossible for these devices to support a wide range of DTLS extensions and cipher suites (e.g., only one suite might be supported). This also means that verbose operations such as checking certificate revocation lists or performing OCSP [21] checks typically cannot be supported.

Powerful Internet hosts on the other hand may expect constrained devices to support security features similar to those found on the conventional Internet (e.g., with strong authentication and key agreement schemes). As constrained devices cannot support these features (see above), an alternative is to consider third party systems (e.g., middleboxes or off-path systems) that offer such features on behalf of constrained devices. However, in this case, a big issue with conventional end-to-end security is that as the connection is secured end-to-end, a third party is excluded from the communication. Thus, an important question addressed by this work is how third parties can take part in securing (but also optimizing; see later) communications with constrained devices in order to bridge the gap with powerful Internet hosts.

While DTLS can avoid the TCP handshake, it still has to perform its own handshaking mechanism in order to negotiate key exchange and authentication methods. The overhead of this handshake in terms of delay or amount of network traffic can be problematic for some types of constrained nodes and networks. Specifically, previous research has shown that in duty-cycled multi-hop networks, the delay introduced by the DTLS handshake can run up to fifty seconds [8] for four wireless hops. The authors also correctly conclude that the memory for storing the DTLS session state on constrained nodes is typically limited to a handful of nodes for Class 1 devices. Additionally, other research [22] has shown that ephemeral DTLS sessions with constrained devices should be avoided as their energy expenditure is up to 60% higher when compared to a single DTLS session with a long lifetime. Therefore, one goal of this work is to limit the impact of the DTLS handshake on delay and energy expenditure, while supporting more than just a handful of simultaneous DTLS sessions per constrained device.

The third group of issues stems from naively deploying end-to-end security in (multi-hop) Low-power and Lossy Networks (LLNs) and from allowing unmonitored access to LLNs to malicious users. In these networks, resources are sparse (see above), and care should be taken in order to avoid unwanted depletion of these resources by Denial-of-Service (DoS) attacks. For example, by repeatedly opening and closing DTLS sessions, a malicious user can significantly reduce the lifetime of a battery-powered device. A malicious user could also send large datagrams to the LLN, which will trigger fragmentation that can exhaust the allocated network buffers in the LLNs. Most of these resource-depletion threats can be mitigated by monitoring and restricting access to the LLN at the edge of the network, where an unconstrained firewall or gateway system resides. However, end-to-end security encumbers such systems from authenticating parties (as constrained devices cannot support strong authentication) and therefore restricting access to authorized parties. Here, this work will study how end-to-end security can be reconciled with the need for traffic filtering at the edge of the network and the need for strong authentication.

### 3.2. Complex Application Features in LLNs

Apart from security issues, there is another important category of problems that relate to the functionality at the application layer for constrained devices, which is targeted by this work. Firstly, the same constraints that prohibit offering extensive security features also apply to implementing application features on the constrained device. This is one of the reasons why the IETF has standardized special purpose protocols and data formats for use in constrained environments (e.g., CoAP and CoRE Link Format (CLF) [23]). However, traditional Internet hosts do not always implement these protocols and data formats. In these cases, a protocol and data format translation should occur that enables the Internet host to communicate with the constrained device (e.g., an HTTP/CoAP proxy and a JSON/CLF mapper). Such a translation has to be performed by an unconstrained third party system (e.g., gateway). Secondly, some types of functionality can be ineffective when they are offered on the constrained device. An example is caching the responses of a constrained server on the device itself, which will not save any network traffic. A second example is the aggregation of observation relationships by intermediaries; clearly, this has to be offered on an intermediary and not on a constrained node in order to have any effect. Note that conventional end-to-end security does not allow for response caching or observation aggregation, as all traffic passing at an intermediary is encrypted. Thirdly, some functionality can be inefficient when they are implemented on the constrained device. An example is storing verbose semantic descriptions on a constrained device, which will lead to significant amounts of network traffic every time these descriptions are requested. Another example of functionality that is inefficient to offer on constrained devices is access control. Typically, the LLN will have already spent a significant amount of resources delivering the request to its destination where it will end up being discarded. Clearly, discarding this request before the network has wasted its resources is more efficient. For these cases, this work will study how third party systems can support and optimize the operations of constrained devices and LLNs.

### 3.3. Problem Statement: Illustration in a Smart Building Use Case

**Figure 3:**
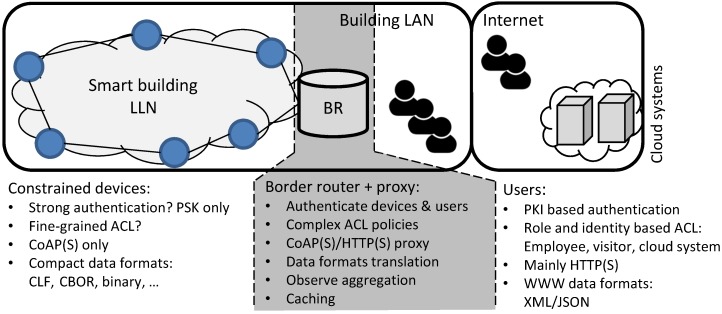
In a smart building scenario, there is a wide variety of different users. Constrained devices are unable to offer all necessary security and application features to cater to these users. In the approach followed by this work, unconstrained systems (e.g., border routers (BRs)) assist by offering these missing features. CBOR: Concise Binary Object Representation, ACL: Access Control List.

Figure 3 shows a smart building scenario that illustrates the problems targeted by this work. In a smart building most of the building services can be monitored and controlled over the Internet. Such services include for example the management of doors, lighting, climate control (e.g., AC), elevators and the monitoring of presence in certain areas. Smart buildings, such as offices and public buildings, typically have a large variety of users: visitors, cleaning staff, technicians, employees, etc. Similarly, there are also a number of computer systems that interact with the smart building: e.g., systems for HVAC, surveillance, facility management, etc. Each of these actors accesses the services offered by the building according to specific access control rules that depend on the role and or identify of the actor, e.g., the HVAC system can control the air conditioning units, but cannot control the doors. However, the HVAC system might be allowed to monitor the status of a door adjacent to an AC unit without being able to (un)lock it. Considering the limited resources of constrained devices (see above), managing and enforcing which actions an actor is allowed to perform depending on their role or identity quickly become too complex for the constrained devices. Furthermore, as most constrained devices only support PSK-based authentication, such a system would require management of shared secret keys between every two actors. Limitations on the LLN and the constrained devices also prohibit these devices from offering protocols and data formats that are common to the unconstrained actors, such as HTTP(S) and XML/JSON. The gray center of the figure already hints at our approach detailed in the next section: a proxy offers many of the missing features on behalf of the constrained devices.

Finally, one might question why this work relies on end-to-end security via DTLS at all, when there appear to be many problems in constrained environments according to the discussion above. Our main motivations for doing so is that DTLS is a proven (and secure) standard, is widely available, is commonly used on the Web and is standardized for use with CoAP. Alternatives to DTLS are either proprietary, or still in the process of standardization (e.g., Object Security of CoAP (OSCOAP) [24]), not applicable to constrained environments (e.g., network layer security), or cannot provide the same level of security as DTLS (e.g., physical layer security). Object security specifically can be considered complementary to transport layer security, and while it is not considered in this work, it can be combined with the work presented here (if feasible given the constrained environments under consideration). The Related Work section discusses object security in greater detail. While the literature shows that lightweight network security is feasible in constrained environments (e.g., compressed Internet Protocol Security (IPsec) [25]), it is not considered in this work because CoAP standardized end-to-end security over DTLS as its security mechanism.

## 4. The Secure Service Proxy

The approach followed in this work allocates one reverse CoAP(s) proxy per constrained device. The CoAP specification [9] defines a reverse proxy as “an endpoint that stands in for one or more other server(s) and satisfies requests on behalf of these, doing any necessary translations”, and it also states that “The client may not be aware that it is communicating with a reverse-proxy; a reverse-proxy receives requests as if it were the origin server for the target resource.” The reverse proxy approach enables splitting the end-to-end communication between a constrained device and its client at the proxy with no need for any additional configuration on the client (as mentioned in the CoAP specification). While the resulting communication is no longer end-to-end, indeed the proxy will share DTLS security contexts with both parties and will translate CoAP messages, the resulting system has many benefits and is able to overcome all of the issues that are discussed in the previous section. Additionally, our reverse proxy approach implements a virtual device for every constrained device. This enables the reverse proxy to extend a constrained device (beyond only proxying) by hosting functionality on the corresponding virtual device. Finally, by enabling the reverse proxy to be deployed on any system (see design), it is not restricted by the limitations common to constrained IoT devices. In the next subsections, we argue that the benefits of this approach far outweigh the downsides of splitting the end-to-end communication, and we present our design for such a reverse proxy.

### 4.1. Motivation of Approach

Our motivation for following a reverse proxy approach consists of two facets: one for the security-related aspects of constrained devices and LLNs and one for the application layer-related aspects of constrained devices. In terms of security, the reverse proxy approach allows one to setup two sorts of DTLS sessions: “lightweight” sessions between the constrained devices and their reverse proxy and fully-featured sessions between the proxy and the clients of the devices. The lightweight sessions employ security primitives that are known to the constrained devices (e.g., pre-shared keys for authentication and key exchange), while the fully-featured sessions can use conventional security methods that are known to the clients: e.g., certificates for strong authentication and Elliptic Curve Diffie–Hellman (ECDH) for the key exchange (including ephemeral key exchanges if perfect forward secrecy is required). Additionally, the reverse proxy can be configured to maintain one long-term session with the constrained device while simultaneously keeping active sessions with multiple clients. This allows one to overcome the small session pool at the constrained devices (due to its limited memory, see above), as well as limit the total number of handshakes performed by the constrained device during its lifetime. As a result, the impact of the DTLS handshake on the LLN and the communication in terms of, e.g., traffic and communication latency is lowered. Finally, the reverse proxy also protects the LLN from a number of resource depletion attacks from attackers on the Internet. By design, a reverse proxy handles all messages for all constrained devices in an LLN from Internet hosts. Thus, the reverse proxy becomes the main traffic entry point for the LLN, and therefore, it can inspect, filter and drop traffic in order to root out traffic from malicious users. Combined with the strong authentication of clients and an access control policy, this proxy can make more informed decisions in regards to filtering traffic when compared to, e.g., a simple Internet firewall.

In terms of the application layer, a reverse proxy is free to process and transform the requests it receives from clients as it chooses. A reverse proxy can improve network access by offering features such as caching, network-edge access control and enforcing congestion control algorithms. Interoperability with other systems can be increased by, e.g., translating between HTTP and CoAP, which is fairly straightforward considering the design goals of CoAP. Translation between different data types (e.g., CoRE link format [23] to JSON) can also boost interoperability. Such a proxy can also implement additional application functionality on behalf of the constrained device. Examples of such functionality include extending the constrained device with semantic descriptions for its resources, a deployment location photo, the weather near the device, etc. Additionally, a proxy can choose to facilitate adding, configuring and deploying such functionality via a plugin-like system. This greatly eases the management of such functionality at run time by making adding, updating, enabling and disabling such functionality easier.

It is important to reiterate that all of the above is possible without any additional configuration on either the constrained device or the client; nor does the presented approach require any modifications to the standards compliant protocol stacks (e.g., 6LoWPAN/DTLS/CoAP) running on the constrained device and the client. Indeed, the client discovers the Internet endpoint of the constrained device that is hosted on the proxy, and the proxy takes care of mapping every request to the corresponding constrained device. In the scenario presented here, all configuration is limited to the proxy. These last two benefits are an important differentiator from existing work, as will be discussed in the Related Work section.

While the reverse proxy approach offers a number of benefits, it also entails some risks that if ignored might undermine the presented system. One risk is that the reverse proxy presents a single point of failure in terms of security and operation. Indeed, if the reverse proxy were to be compromised then, e.g., all session keys and long-term keying material (pre-shared keys and private keys) could be made public. As the proxy offers a RESTful interface for managing virtual hosts and their keying material, this interface entails a security risk and should therefore be properly hardened against malicious usage (see Section 4.3.1 for suggestions). Likewise, if the reverse proxy were to be the target of a resource depletion attack, then the constrained devices hosted by that proxy would become unreachable. On the other hand, as the proxy is deployed on a more powerful system, the proxy is more resilient to resource depletion attacks than constrained devices and networks. A second issue is the introduction of a third party (i.e., the proxy itself) into the trust model by terminating the end-to-end security that must be trusted by both the constrained device, as well as the clients. As all collected data and issued commands pass via the proxy, this can raise privacy concerns when the device or the client does not trust the owner of the proxy. One option to mitigate this privacy risk is to let the owner of the constrained devices operate the reverse proxy on his or her own. To this end, our evaluation shows that a low-cost single board computer (e.g., Raspberry Pi) is capable of hosting the proxy, which enables on-premises deployments. To summarize, the proxy breaks end-to-end security in order to provide additional features, which address operational and performance concerns of resource constrained devices. This work argues that the benefits of terminating the end-to-end security outweigh the security-related risks in the case of ’Class 1’ resource constrained devices and networks. For less constrained devices and networks, this balance might tip in favor of end-to-end security.

### 4.2. Secure Service Proxy: Design

**Figure 4:**
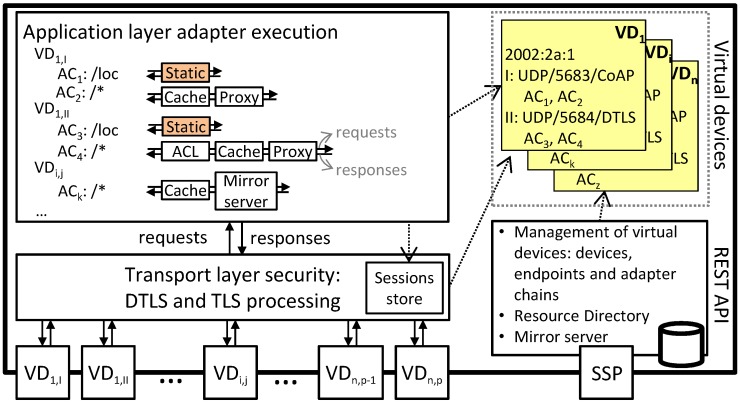
Secure service proxy: design.

In order to enable our proxy to extend constrained devices with a wide range of functionality, the design adopts the concept of virtual devices. In our design, every virtual device is allocated a dedicated IPv6 address from an IPv6 subnet that is either routed to the proxy or directly connected to the proxy. Every virtual device has one or more endpoints associated with it. An endpoint corresponds to a transport and application layer binding: e.g., UDP/CoAP, DTLS/CoAP, TCP/HTTP or TLS/HTTP. For every virtual device, the proxy listens for traffic on each of its endpoints; this is shown in the bottom left of Figure 4.

The transport layer security block is responsible for handling the (D)TLS protocol for secure endpoints on behalf of virtual devices. As such, this block performs (D)TLS handshakes, thereby authenticating the client and performing a key exchange. To this end, the block interfaces with the virtual device configuration (top right in the figure) to retrieve the TLS parameters that are configured for the virtual device. These parameters include a list of available cipher suites and keying material for the secure endpoint of the virtual device, as well as whether the virtual device requires clients to authenticate themselves. Apart from the handshake, this block is responsible for tracking active sessions with virtual devices (via the sessions store). It also decrypts and verifies incoming (D)TLS application data messages, which are passed on to the adapter execution block, as well as encrypts outgoing application data that come from the adapter block. The keying material and the protocol state used in the encryption and verification process naturally depend on the endpoint involved.

Incoming messages contain (secured) requests, which are either HTTP or CoAP requests. While our design supports adapters for both application layer protocols, we foresee that HTTP requests will almost always be translated immediately to a CoAP request. As such, we do not expect virtual devices to host only an HTTP endpoint (although the design does support this). When the application layer adapter execution block receives a request, it will search through the tree of available adapter chains to search for a chain that is the most specific match for the request. The current implementation supports searching based on the address and endpoint of the virtual device, as well as the URI of the request.

Once a chain has been found, the execution block will pass the request along the chain. Every element of the chain (i.e., an adapter) can either return (a modified) the request, which will be passed to the next adapter in the chain, or stop the execution of the chain by returning a response. The current implementation allows returning a response from an adapter in a non-blocking (i.e., asynchronous) way, as retrieving a response might involve a lengthy IO operation. Once the response is available, it is passed along the chain in reverse. This allows adapters to process and (if needed) modify the response before it is stored in the virtual device and returned to the client.

Application layer adapters implement the functionality hosted by virtual devices. The idea underlying adapters is to compartmentalize functionality into modules that can be reused by virtual devices. When creating an adapter chain, an instance for every adapter in the chain is created, and every instance is configured according to the parameters exposed by the adapter type (see further). While instances of adapters reside in adapter chains, they can be shared by more than one adapter chain (AC). For example, in Figure 4, the same Static adapter instance (colored orange) is shared by ${\mathrm{AC}}_{1}$ and ${\mathrm{AC}}_{3}$. This is mainly useful when the same functionality should be available for multiple endpoints of the same virtual device (e.g., CoAP and CoAPs) or when an adapter implements functionality that does not require configuration that differs per adapter chain (e.g., a logging adapter that logs all incoming requests for auditing purposes).

The proxy also exposes a networked interface in the form of a REST API to manage virtual devices, which is shown in the bottom right of Figure 4. The REST API allows creating and deleting virtual devices and their endpoints, as well as instancing and deleting adapters and defining adapter chains. When creating (D)TLS endpoints, the REST API also allows specifying the cipher suites supported by the virtual device, as well as the keying material (e.g., X.509 certificate or private key). Apart from the management interface, the proxy also hosts a resource directory that contains the hosted virtual devices. Finally, a mirror server is also available to enable resource updates from constrained devices that are asleep for continuous and long periods of time (i.e., sleepy devices). This mirror server can be used by virtual devices to interface with resources from sleepy constrained devices.

Finally, the presented design allows one to deploy the proxy on different locations in the network by varying the IPv6 subnet for the allocation of virtual device IPv6 addresses. We foresee two scenarios. In the first scenario, the proxy resides close to the constrained devices by allocating addresses from a neighboring LAN network to virtual devices. An example would be a home LAN network from which the proxy assigns unused addresses to virtual devices. In the case of a 6LoWPAN network, the proxy can be combined with the border router. This scenario also aligns nicely with the distributed computing concept that is commonly found in fog computing and in in-network processing [26]. In a second scenario, the proxy resides further ’upstream’ from the constrained devices (e.g., in a data center, the cloud, etc.) and allocates addresses from a special-purpose IPv6 subnet that is dedicated to virtual devices. In this scenario, the routing has to be configured to route this special-purpose IPv6 subnet via the proxy (which is not a problem in most data centers). Both scenarios are complementary and will depend on the specific needs of the considered use-case: e.g., a proxy in the LAN network means that data stay inside the home network, which may benefit privacy. Similar considerations were previously discussed in the problem statement section.

### 4.3. Secure Service Proxy: Implementation

For the implementation of our secure service proxy, we chose to build upon the previous work in our CoAP++ framework (which in turn builds on top of the Click modular router software). This choice provides a great amount of flexibility in how we process the network traffic for the virtual (and constrained) devices, as all routing functions are part of Click and can therefore be configured to our liking. In terms of the (D)TLS implementation, we chose to use the wolfSSL library as this offers the easiest API for managing sessions and integrating into the Click router where most processing happens on network packets.

#### 4.3.1. Virtual Devices and Endpoints

Virtual device endpoints are created and deleted via the management interface. This is a straightforward REST interface that is hosted on the secure service proxy over CoAPs. As this interface handles sensitive information such as keying material, access is restricted to authorized users, which are allowed to manage endpoints and adapter chains.

POST requests with an endpoint description are used to create a new endpoint for a virtual device. The endpoint description contains both the virtual device to which the endpoint belongs, as well as any configuration details describing the endpoint itself. This description is serialized as a JSON object in the payload of the POST request. For a plain-text CoAP endpoint, the configuration details are limited to the UDP transport port of the endpoint. For a DTLS CoAPs endpoint, the configuration also includes information about the supported cipher suites and any parameters for the cipher suites. In the current implementation, the “TLS_PSK_WITH_AES_128_CCM_8” and “TLS_ECDHE_ECDSA_WITH_AES_128_CCM_8” cipher suites are supported for CoAPs endpoints. When creating an endpoint that supports the PSK cipher suite, the pre-shared-key and an (optional) client identity hint have to be specified as parameters. For the elliptic curve DSA suite, the secp256r1 private key and signed certificate have to be provided as parameters. These are both encoded in base64 in the endpoint description. The following listing contains an example POST request that creates a CoAP endpoint for a virtual device hosted under 2001:6a8:1d80:23::1 on port 5684 with an ECC cipher suite.

POST /virtualDevices
Content-Format: application/json
{
  "address": "2001:6a8:1d80:23::1",
  "prefixLen": 128,
  "port": 5684,
  "dtls": {
    "supportedCipherSuites": [
        {
          "cipherSuite": "TLS_ECDHE_ECDSA_WITH_AES_128_CCM_8",
          "parameters": {
             "b64PrivateKey": "QVNO...==",
             "b64Certificate": "LS0t...=="
           }
         }
    ]
  }
}
 
2.01 Created /virtualDevices/2001:6a8:1d80:23::1~128~5684
          

The response of the secure service proxy links to a newly-created resource that can be used to delete the endpoint at a later time. This resource is also used for managing the adapter chains that belong to an endpoint, as explained in Section 4.3.3.

#### 4.3.2. Implemented Application Layer Adapters

In terms of application layer adapters, our proxy currently implements the adapters listed in Table 1. This section describes each of the adapter types in more detail.

The access control adapter applies Access Control List (ACL) rules to the CoAP(s) requests it processes. ACL rules are parsed as JSON objects that assign allow and deny rules to either a username or a role of users. An allow and deny rule consists of a regular expression, which is applied to the request URI, and a list of request methods. In case no matching ACL rule is found, then the default policy of the adapter instance (either accept or deny) is applied. The following JSON serialization of an example ACL rule gives user “bob” full access to the devicename resource, while access to the lock resource is restricted to read only.

{"username": "bob",
"allow": [{"uri-regex":"devicename", "methods":["GET", "PUT", "POST", "DELETE"]},
    {"uri-regex":"lock", "methods":["GET"]}],
"deny": []}
          

Hosting a virtual resource on a virtual device is the task of the static resource adapter. In order to allow arbitrary content types of the payload, the value of the virtual resource is encoded in base 64 in the configuration of the adapter. An example is shown in the next section.

The cache adapter serves and caches responses for requests to virtual devices. The cache adapter calculates a cache key for every CoAP request it handles. When a fresh response matching the cache-key is found, the adapter chain’s execution is halted, and the cached response traverses the adapter chain in reverse. Responses processed by the cache adapter are handled in accordance with Section 5.9 of the CoAP RFC [9]. This means that, e.g., a “2.05 Content” response will be cached, while a “2.04 Changed” response will mark any stored response as not fresh. Cached responses are removed when they expire after their Max-Age option. Note that the cache adapter does not implement the “Validation Model” specified in Section 5.6.2 of the CoAP RFC [9]. When used in conjunction with access control, it is important that all ACL rules are applied before hitting the cache, as the execution of the request leg of the adapter chain will stop when a cache hit is found. The underlying implementation caches responses in memory via a memcached instance.

The congestion control adapter in its current form applies traffic shaping on a per host basis. Currently, it is possible to limit the number of open requests between a client and a specific virtual device and between a client and a group of virtual devices. This group encompasses all virtual devices with an adapter chain that shares the same congestion control adapter instance. Open requests are requests for which a response has not been sent yet. If a client reaches its limit, then the request is dropped until either a response is received or one of the prior requests of that client is removed after a time out period (can be configured). Finally, a client can either be identified by its endpoint address or by its identity derived from the authentication credentials during the (D)TLS handshake.

The .well-known/core adapter is responsible for including the functionality that is hosted on the virtual devices in the resource discovery responses of the real constrained device. In the current implementation, the .well-known/core (wkc) adapter asks every adapter from all of the adapter chains that are defined for the virtual device to modify the discovery response from the real device. This way, the static resource adapter can add a link to its virtual resource, and the ACL adapter can remove links for resources that the user is not authorized to access. To this end, every adapter type offers a “processDiscoveryResponse” method that is used by the wkc adapter.

The proxy adapter takes a request for a virtual device and issues a new CoAP request to the corresponding actual constrained device. Therefore, an instance of this adapter is configured with the CoAP(s) endpoint of the constrained device. Only the transport layer addresses are changed; the new CoAP request is copied from the output of the previous adapter in the adapter chain (with the exception of the message ID and the token, of course). The proxy adapter will either retrieve a response or generate a time-out; therefore, it always comes last in adapter chains. This adapter will also combine observation registrations when it receives multiple registrations for the same resource on a virtual device. Likewise, it also multiplexes responses from constrained devices to multiple clients in case there is more than one ongoing observation registration.

Finally, the mirror server adapter is a special type of proxy adapter in that it issues CoAP(s) requests to a mirror server instead of the constrained device itself. Apart from the end point of the mirror server, also the handler of the constrained device is configured into the mirror server adapter instance. For instance, a request to the coaps://vd1.iot.test/status resource on a virtual device would be translated to coaps://ms.iot.test/ms/0/status.

#### 4.3.3. Adapter Chain Management: Interface

Once an endpoint for a virtual host has been allocated on the proxy, adapter chains can be created and hosted on that endpoint. Building on our previous example, the listing below contains a CoAP request that instantiates an adapter chain, which contains the access control, well-known core rewriting, caching and forward CoAPs proxy adapters. Again, the payload is a JSON object that describes the chain and contains the parameters for the different adapter instances. The adapter chain is created as the default chain via the wildcard character in the chain path. The default chain is executed for requests where no other adapter chains with a matching URI path are found.

POST /virtualDevices/2001:6a8:1d80:23::1~128~5684
Content-Format: application/json
{
    "path": "/*",
    "pipeline": [
      {
      "type": "acl",
      "default_access_control_policy": "deny",
      "rules": [
         {"username": "fvdabeele", "rules": [{"uri-regex":"regex1", "allowMethods":["*"]},
                               {"uri-regex":"regex2", "allowMethods":["GET"]}]},
         {"username": "*", "rules": [{"uri-regex":"regex1", "denyMethods":["*"]},
                 {"uri-regex":"regex2", "allowMethods":["GET"]}]}
       ]
    },
    {
       "type": "wkc"
    },
    {
       "type": "cache",
       "default_lifetime": 60
    },
    {
       "type": "proxy",
       "scheme": "coaps",
       "addr": "bbbb::1",
       "port": 5684
    }
  ]
}
 
2.01 Created /virtualDevices/2001:6a8:1d80:23::1~128~5684/*
          

The second example, shown in the listing below, details how a static resource is created on the endpoint of our virtual host (in this case, it contains a semantic description of the virtual host in the RDF format). The chain also illustrates the linked adapter, which refers to the ACL adapter instance that was created in the previous listing. The link points to the management resource of the adapter instance.

POST /virtualDevices/2001:6a8:1d80:23::1~128~5684
Content-Format: application/json
{
    "path": "/rdf",
    "pipeline": [
    {
      "type": "linkedAdapter",
      "link": "/virtualDevices/2001:6a8:1d80:23::1~128~5684/*/0"
    },
    {
      "type": "static",
      "contentType": 41,
      "value": "PGh0d...=="
    }
  ]
}
 
2.01 Created /virtualDevices/2001:6a8:1d80:23::1~128~5684/rdf
          

Finally, note that the parameters of existing adapters can be updated via a PUT request to the management resource of the adapter instance. In this case, the payload is a JSON object where the keys are the parameter names. Likewise, adapters and chains can be deleted via their respective management resources.

#### 4.3.4. Authenticating (D)TLS Clients on the SSP

In order to facilitate the authentication of users and the authorization of user actions, the SSP links client authentication information (e.g., TLS PSK or X.509 client certificate) with users and roles. The current implementation is limited to using TLS primitives for supplying authentication credentials, although in the future, alternatives might be considered (e.g., lightweight application-layer access tokens). For example, a (D)TLS session that was setup with ${\mathrm{PSK}}_{1}$ as the pre-shared key can be linked with ${\mathrm{user}}_{A}$. Likewise, attributes in a client X.509 certificate that is signed by a party trusted by the SSP can be linked with a specific user, e.g., a certificate issued by ${\mathrm{CA}}_{A}$ with the common name attribute set to *fvdabeele* can be linked with ${\mathrm{user}}_{B}$. Finally, the proxy also exposes a RESTful interface for managing which credentials belong to which user and the roles of users.

#### 4.3.5. Key Management between SSP and Constrained Devices

The SSP contains an in-memory repository of pre-shared keys and corresponding identity hints in order to setup DTLS sessions with resource-constrained CoAPs servers. As this repository contains all of the keying material for the constrained devices known to the proxy, it contains sensitive information and should be handled accordingly. In the current implementation, this repository is initialized when the SSP process is started. A future extension could enable at run-time manipulation of this repository by, for example, specifying keying material when instantiating coaps proxy adapters. Currently, this has not yet been implemented, as in our use cases, this repository does not change frequently and remains stable. In use cases where the repository is more volatile, such an extension could enable better key management.

## 5. Related Work

The concept of device virtualization in the IoT is widespread in the literature, though often times under different names such as sensor, thing and object virtualization. Indeed, in [27], the authors present a survey on object virtualization in the IoT stating that “the concept has become a major component of current IoT platforms where it aids in improving object energy management efficiency and addressing heterogeneity and scalability issues”. The authors classify existing architectures as one or many real objects for one or many virtual objects. While the focus in this work has been on one real object for one virtual object, the flexibility of the presented design enables the same adapter to be shared by multiple virtual devices, as well as one virtual device to span multiple physical devices (for example, a virtual device combining all lamps in a room).

There exist numerous works in the literature that study the benefits of using third parties or intermediaries in constrained environments. In order to narrow the scope of this section, only works that are relevant in the context of constrained RESTful environments are discussed here. In [28], Kovatsch et al. discuss moving application logic from firmware to the cloud. According to the vision of the authors, devices are thin servers exposing RESTful resources for data access and actuation, and most of the application logic would reside in the application servers. While our approach also advocates thin servers for devices, deploying the SSP in the cloud is optional. In use-cases where local access is important, the SSP may reside closer to the devices (e.g., deployed in the LAN) in order to meet requirements with respect to latency, privacy or availability. Additionally, the SSP may support constrained nodes and applications servers by providing functionality such as caching and more scalable authentication and authorization. The IPv6 addressing proxy presented in [29] is an example of an intermediary system for mapping legacy technologies to the IPv6 Internet of Things. By allocating IPv6 addresses to map to different legacy technologies, the approach is similar to the virtual devices presented in our work. Note that the adapter concept provides the flexibility to map virtual devices to different technologies similar to the work in [29]. While not presented in this work, the SSP has been used to host LoRaWAN end devices as virtual IPv6 CoAP endpoints via an Advanced Message Queuing Protocol (AMQP) publish/subscribe adapter that interfaced with the LoRaWAN network server. The authors in [30] propose to interconnect Web applications based on HTTP and Web sockets with CoAP-based wireless sensor networks via a CoAP proxy. The CoAP proxy focuses on translating between different protocols and closely follows the guidelines outlined in RFC 8075 [31]. The scope of the SSP is broader, as it includes transport security, access and congestion control next to mapping HTTP to CoAP. Finally, note that the forward proxy approach of Ludovici differs from the reverse proxy approach of the SSP. In [32], Mongozzi et al. introduce a framework for CoAP proxy virtualization in order to address the scalability and heterogeneity challenges faced in large-scale Web of Things deployments. The framework installs a reverse CoAP proxy on the sensor network gateway and then applies virtualization so that the proxy can be customized and extended by third parties without modifying the reverse proxy. All interactions of these virtual proxies with smart objects pass via this reverse proxy, which acts as an arbiter for access to the limited resources of the smart objects. The presented approach is interesting as the containerization of the virtual proxies into virtual machines makes them more flexible than the adapter approach followed in the SSP. We have experimented with providing some degree of extensibility by creating adapters from python scripts in the SSP (these scripts could be uploaded via the adapter chain management interface). While this python adapter type provided some degree of customization, the lack of proper process isolation meant that (malicious) scripts could stall the SSP. As such, these python adapters did not make the final SSP design. While the concept of the virtual proxies is interesting, the extent of the work is limited as the focus lies on the virtualization technique, and interesting features such as scalable security and efficient and authorized network access are not considered. Instead, the authors focus on providing service differentiation between multiple virtual proxies. Also note that proxy virtualization is not the same concept as device virtualization, though they can be used to solve similar problems. The same authors of [32] look at the specific problem of proxying CoAP observation efficiently for different QoS requirements in [33]. While the scope of the work is quite different from this paper, the use of a reverse proxy for bundling observation relationships is shared between the two works. Another example of device virtualization in RESTful environment is [34], where the authors assign virtual coap servers to RFID tags. The actual CoAP servers are not running on the tags though. Instead, they reside on RFID readers, which are able to enhance tags with additional functionality (such as discovery). This work has parallels with the SSP, which enhances constrained devices by means of application layer adapters.

A second category of relevant works in the literature studies the challenges faced by transport layer security in constrained IoT environments. There are a number of works that study the DTLS handshake, as it is a fairly complex and verbose process with significant resources requirements for constrained devices. In [35], Hummen et al. propose a delegation architecture that offloads the expensive DTLS connection establishment to a delegation server, thereby reducing the resource requirements of constrained devices. The delegation architecture also enables more complex authorization schemes, as it has more resources at its disposal. The authors report significant reductions on memory overhead, computations and network transmissions on constrained devices. Our termination method can also provide complex authorization schemes of the virtual device. In Section 6.1, we have also reported significant savings in regards to CPU and network resource usage (and consequently, energy usage). While our approach still requires an active DTLS session between the SSP and the constrained device, the number of handshakes during the lifetime of a device is drastically reduced. While the memory requirements are not as low as in [35], they are still lowered as the constrained device can limit the number of simultaneous sessions to one. Finally, note that our approach does not require any changes to the DTLS stack running on the device. The work in [36] focuses on various challenges in deploying DTLS in resource-constrained environments. Similarly to [35], the approach revolves around handshake delegation. The authors adopt the concept of secure virtual things in the cloud where physical things delegate the session initiation to their corresponding virtual thing. As a result, physical things can limit their DTLS implementation to only the record layer protocol, which leads to drastic memory savings. One interesting aspect of the presented architecture is that the physical thing can assume both roles of client and server. Unfortunately, the concept of virtual things is not extended beyond the handshake delegation mechanism. It would be interesting to combine a delegation mechanism with some of the findings presented in our work. A hybrid option would be possible where the delegation mechanism is used for the most constrained devices (requiring a custom lightweight DTLS stack) and where the termination mechanism can be used for devices with sufficient memory (i.e., where a full DTLS stack is feasible) or where the DTLS stack cannot be customized to implement the delegation method.

Object Security of CoAP (OSCOAP) [24] is an IETF Internet Draft standardizing end-to-end security of CoAP options and payload at the application layer. While the specification focuses on the forwarding case when using a forward proxy (which excludes caching), it does include an appendix describing a mode of operation, Object Security of Content (OSCON), which is compatible with caching responses at intermediaries. The draft notes that OSCOAP may be used in extremely-constrained settings, where CoAP over DTLS may be prohibitive, e.g., due to large code size. Nevertheless, the authors state that OSCOAP may be combined with DTLS, thereby benefiting from the additional protection of the CoAP message layer present in DTLS-based security. Note that the standardization efforts focus on the case of a forward proxy, whereas this work focuses on a reverse proxy approach. As such, the trust models are different as the reverse proxy represents the end device from the point of view of the client. Despite the difference in proxy models, the two approaches remain compatible and could strengthen each other. For example, the SSP could implement OSCOAP for cases where clients are employing a forward proxy, which is not trusted by the client. Additionally, it would be interesting for the SSP to support OSCOAP as a lightweight alternative for DTLS to protect communications with constrained devices with severe memory limitations. In such a case, clients would communicate securely with the SSP over DTLS while the communications between the SSP and the constrained devices would be protected either via OSCOAP (e.g., for constrained devices with severely limited memory) or via DTLS (e.g., for constrained devices with sufficient memory).

Finally, in high volume Web environments, transport layer security is often terminated at a proxy deployed close to the Web server(s). The main motivation for terminating TLS is that it enables load balancing, where terminated HTTPS requests are distributed over multiple Web servers. Load balancing increases the availability of the Web deployment, as the outage of one Web server does not affect the service availability in this case. Popular Web proxy software, like nginx and HAProxy, supports different reverse proxy deployment options for terminating TLS. Similarly, the elastic cloud computing platform of Amazon.com, Amazon Web Services, supports TLS termination and load balancing by virtue of its HTTPS listener service. While the main motivation of the SSP for session termination is not load balancing, the SSP does apply termination in order to be able to move computationally-expensive and verbose operations from constrained devices to the proxy, which improves performance. Similarly to high availability TLS proxies, the SSP may reduce key management complexity, as all keying material for public communications is stored on one system.

## 6. Evaluation: Results and Discussion

This section presents two evaluation scenarios that show the gains attainable by our approach. Such gains include: a decrease in load on constrained devices and the LLN, lower energy usage for constrained devices, an increase in user handling capacity of LLNs, more responsive LLNs, more scalable authentication and better authorization. The evaluation scenarios were chosen to evaluate the impact of the proxy on two specific operational aspects of LLNs: setting up DTLS sessions with constrained devices over multiple wireless hops and observing CoAPs resources on constrained devices from multiple DTLS clients.

### 6.1. Terminating End-To-End-Security at the SSP

The first evaluation scenario is geared towards quantizing the impact of splitting end-to-end security at the smart service proxy. More specifically, the goal is to study the impact of re-using a DTLS session of a constrained CoAPs server on the operation of both the constrained node, as well as the CoAPs client.

#### 6.1.1. Simulation Setup

**Figure 5:**
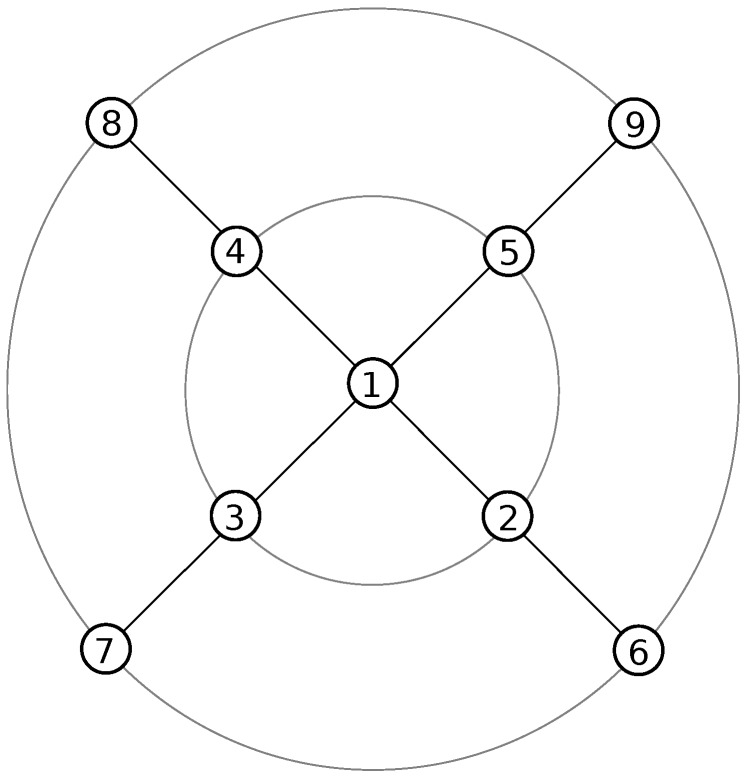
Cooja network topology: four CoAP(s) servers (6, 7, 8, 9) are located two hops away from the IPv6 Routing Protocol for Low-power and Lossy Networks (RPL) border router.

Extensive simulations were performed with a nine-node 6LoWPAN network arranged in a cross topology as detailed in Figure 5. One node is at the center of the cross and is the RPL border router of the 6LoWPAN network; four nodes are intermediate routers (each located in the middle of one of the four legs of the cross); and the last four nodes are CoAP(s) servers that are located at the four ends of the cross. The border router is connected to the smart service proxy, which is running on the same PC as the Cooja simulator. Finally, an unconstrained CoAP(s) client interacts with the CoAP(s) servers. In the evaluation scenario, the client sends the following sequence of CoAP(s) requests: a .well-known/core discovery request, a sensor measurement request for the “/s” resource and an actuator request for the “/a” resource. The constrained CoAPs servers are running er-coap and TinyDTLS (in Contiki) configured to accept the “TLS_PSK_WITH_AES_128_CCM_8” cipher suite with a PSK hint of 15 bytes.

The same request sequence was sent to the CoAP(s) servers for one reference case and three different SSP configurations: Plain Text (PLT), End-to-End (E2E), first Termination (TER1) and *n*-th Termination (TER). In the PLT configuration, all requests are sent over plain text CoAP. This is a reference cases for the other three cases. In the E2E case, all requests are sent over CoAPs without any termination of DTLS sessions at the SSP. In the case of TER1 and TER, all requests are sent over CoAPs, and the DTLS session is terminated at the SSP. For TER1, there does not exist an active DTLS session between the proxy and the constrained node. Therefore, a new DTLS session must be setup between the SSP and the constrained node. For TER, the active DTLS session in the LLN can be re-used, and there is no need to setup a new DTLS session with the constrained node. For all DTLS cases, the DTLS client always sets up a new DTLS session at the start of a request sequence. It also tears down the existing session at the end of every sequence. As such, this testing scenario represents a large number of DTLS clients that would interact with the constrained CoAPs servers over the lifetime of the constrained node. For each configuration, the request sequence was run four hundred times, i.e., one hundred times per DTLS server. All results were obtained using the default CSMA MAC protocol and ContikiMAC RDC protocol as available in Contiki.

#### 6.1.2. Results

**Figure 6:**
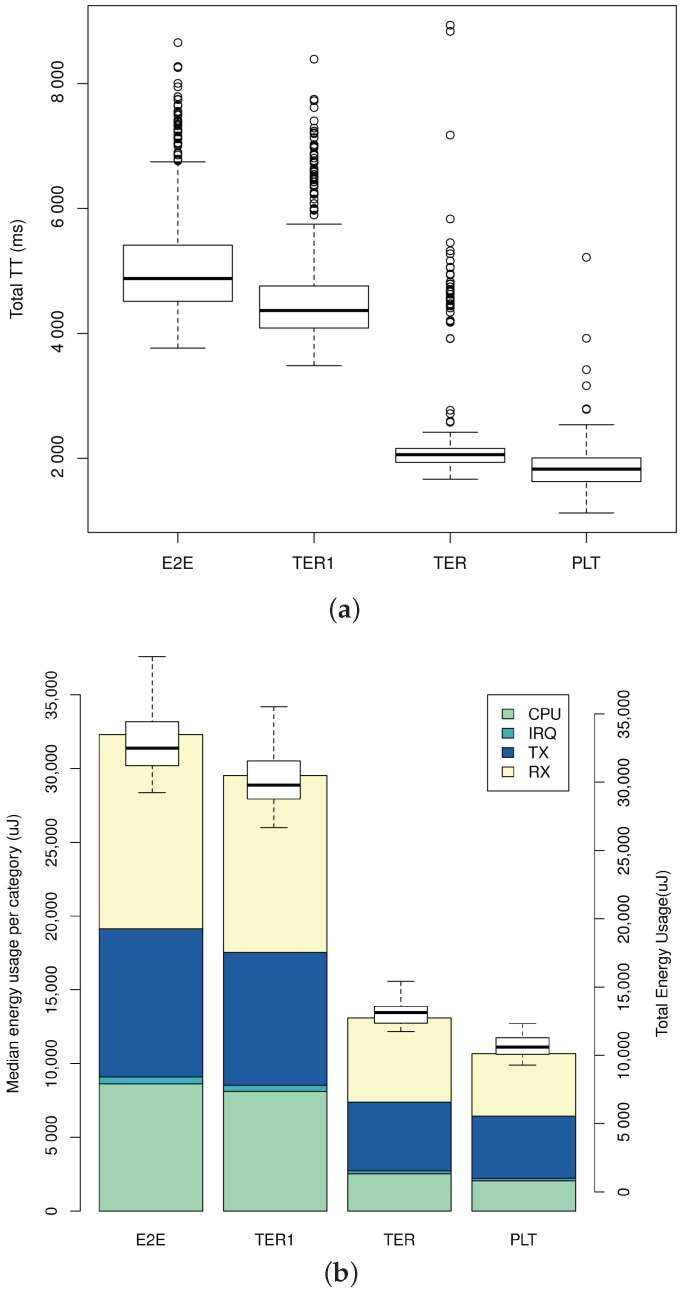
Transaction times and energy usage of the CoAPs servers for the three gateway configurations (End-to-End (E2E), first Termination (TER1), *n*-th Termination (TER)) and the Plain Text CoAP reference case (PLT). (**a**) Total Transaction Times (TTT) for the request sequence; (**b**) median energy usage per category (left axis) and total energy usage (right axis).

Figure 6a shows the Total Transaction Time (TTT). This is the time between the start of the DTLS session handshake (i.e., when the first ClientHello message is sent by the client) and the end of the DTLS session (i.e., when the DTLS Finished message is received by the client). There is a significant reduction in TTT between the E2E and the TER configurations: their medians are 4879 ms and 2060 ms, respectively. This is due to the DTLS session re-use in the LLN, which saves, when comparing the median cases, thirteen packets in the LLN, as the DTLS handshake in the LLN can be avoided in the TER configuration. As a result, the TER configuration is able to closely match the reference plain-text case in terms of TTT. The 233-ms difference in the median is caused primarily by the overhead of the additional DTLS headers. More specifically, the overhead triggers 6LoWPAN fragmentation for the large discovery response in the TER case, whereas this fragmentation is absent in the PLT case.

Figure 6b displays the energy usage for the different configurations. The stacked bar plot shows the median energy usage per category on the constrained device, whereas the box plot shows the total energy usage (to show the dispersion of the measurements). Again, there exist a significant difference between the E2E and the TER configurations: 32,485 µJ vs. 13,133 µJ respectively (a reduction by a factor of 2.4). Similarly to the TTT results, this reduction is primarily due to the absence of the DTLS handshake in the LLN. This is confirmed by the bar plot where the energy usage for the RX and TX categories are reduced the most. The energy consumption in the CPU category is also significantly lower, as the CPU is in low-power mode more often and does not have to perform expensive hash calculations when completing the handshake. All in all, the results allow us to conclude that our approach increases the responsiveness of constrained devices (provided there is an active session in the LLN) while reducing the energy consumption for traffic loads with many DTLS sessions (e.g., traffic loads with many parties).

Finally, it is worth pointing out that our approach drastically limits the total number of handshakes that a constrained node will perform during its lifetime. Apart from the savings discussed above, this also has the additional benefit that, in lossy networks, the total number of failed handshakes will be lower. Indeed, Garcia et al. [37] have shown that in lossy networks, the fraction of failed handshakes can vary significantly based on the packet loss ratio: e.g., 30 to 40% of handshakes fail for a packet loss ratio of ~20%. By limiting the total number of handshakes, our approach also limits the amount of constrained device resources wasted on these failed handshakes. On the other hand, care should be taken to periodically refresh keying material as needed by the underlying cryptographic primitives in use.

### 6.2. Aggregating Multiple CoAPs Clients at the SSP

The second evaluation scenario focuses on the impact of the SSP on constrained devices that serve multiple CoAPs clients simultaneously via CoAPs observation. Unlike clear text CoAP observation, notifications for one CoAPs client typically cannot be reused to serve another client due to the confidentiality of the notification in DTLS. However, the SSP presented in this work can, as a reverse CoAPs proxy, observe one CoAPs resource on a constrained device and use these notifications to serve a multitude of CoAPs clients. The presented evaluation considers up to ten CoAPs clients that observe a resource on a constrained device and compares the case of end-to-end observation versus observation via the SSP. Note that one should keep in mind client authorization when using one CoAPs stream of notifications for serving multiple CoAPs clients, e.g., a client that is not authorized to access a resource on the constrained device must also be denied access to that resource via the SSP. To this end, this work presents and implements an access control adapter, which enforces CoAPs resource-specific access control policies.

#### 6.2.1. Experiment Setup

**Figure 7:**
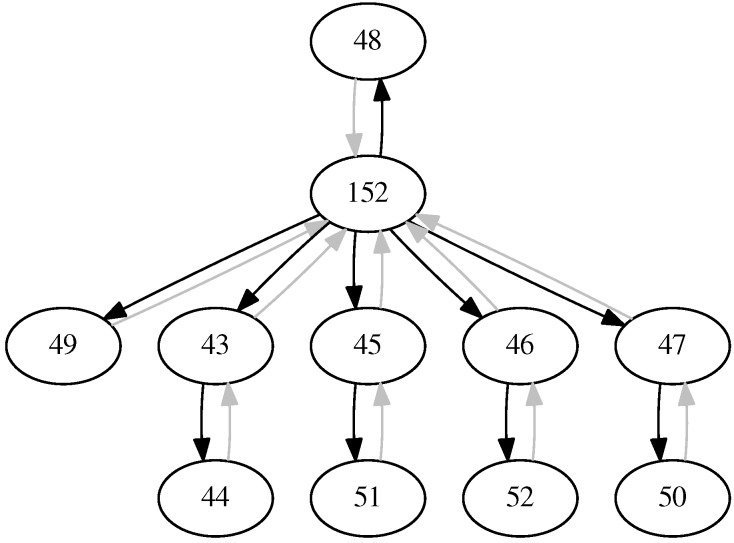
Representative RPL network topology: the node under study, Node #50, is situated two hops from the border router, Node #152.

To quantify the impact of aggregating CoAPs observations at the SSP, a number of experiments were run on a WSN testbed. The experiments consisted of a 6LoWPAN network with ten sensor nodes arranged on a line with six meters of spacing between adjacent nodes. An additional sensor node (Node #152) is situated to the upper left of the line and is connected to a Raspberry Pi 2, where it serves as the RPL border router. The smart service proxy software is running on the Raspberry Pi 2. In order to cope with the changes in the RPL topology between experiments and over time in the same experiment, Node #50 was selected for testing as it was always located two hops away from the border-router. A representative network topology is shown in Figure 7. Note that depending on the experiment, Node #50 might have a different parent than Node #47 (e.g., Node #43 was a common alternative), but in all experiments, there was always an intermediary router between the border router and Node #50.

All wireless sensor nodes employ the msp430f5437 uC with 128 KB of RAM and 256 KB of ROM and the TI CC2520 802.15.4 transceiver. As such, the platform is identical to the WiSMote platform in Contiki in terms of the specifications that are relevant for the presented results. The sensor nodes run a TinyDTLS CoAPs server, which is configured to support three simultaneous DTLS sessions and one simultaneous DTLS handshake. While a binary for four simultaneous sessions could be built, it was not running stably. Attempts to support more than four clients led to a RAM overflow during linking. By default, er-coap in Contiki sends one confirmable notification for every twenty notifications. Finally, all sensor nodes in the network are running the default CSMA MAC protocol and ContikiMAC RDC protocol available in Contiki.

**Figure 8:**
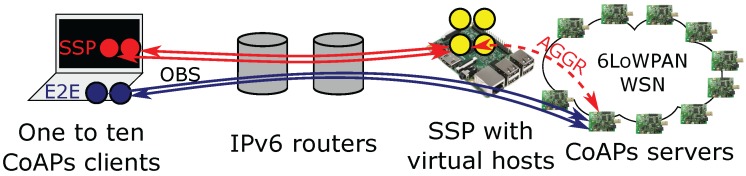
Evaluation setup: a variable number of CoAPs clients observes one of two resources on either the virtual host (SSP) or the sensor node (E2E).

For every sensor node, a corresponding virtual host was created on the SSP. The virtual hosts were configured similar to the listing in Section 4.3.1, with support for the “TLS_ECDHE_ECDSA_WITH_AES_128_CCM_8” cipher suite. This cipher suite provides perfect forward secrecy by means of an ephemeral Diffie–Hellman key exchange between the virtual hosts and the DTLS clients. Additionally, DTLS clients authenticate virtual hosts by means of the x.509 certificates of the hosts, which are signed by a Certificate Authority (CA) trusted by the clients. Similarly, the DTLS clients also present an x.509 certificate during the DTLS handshake, which is signed by a CA that is trusted by the proxy. As a result, the clients may be authenticated at the proxy-side (by mapping attributes from the certificate to a user in the proxy; see Section 4.3.4), which is mandatory for the use of the access control adapter in order to provide fine-grained authorization as presented in Section 4.3.2. Each virtual host was allocated a global IPv6 address from the LAN network of the Raspberry Pi 2 and has one default adapter chain with access control, caching and proxy adapters. The CoAPs clients ran as part of the CoAP++ framework on a PC that was located three IPv6 hops away from the Raspberry Pi 2. All IPv6 addresses in use (i.e., CoAPs clients, RPI, virtual hosts and WSN nodes) were working, global IPv6 addresses. An overview of the evaluation setup is shown in Figure 8.

In all experiments, a number of CoAPs clients observes a resource on either the virtual host or the sensor node. As such, the experiments considered two cases: end-to-end (E2E) CoAPs observations and CoAPs observations via the SSP. In both cases, experiments were run for two CoAPs resources: a resource with a one-second notification period and another resource with a five-second notification period. In the E2E case, experiments were performed with one, two and three simultaneous CoAPs clients. In the SSP case, experiments were performed with one, two, three, four, five and ten simultaneous CoAPs clients. In total, eighteen experiments were performed. Each experiment was run for at least twenty minutes, during which the energy outputs for all sensor nodes were captured every five seconds, and the outputs from the CoAPs clients were stored, as well. This enabled us to quantity the energy usage, as well as the application-layer performance, the results of which are presented in the following section.

#### 6.2.2. Results

**Figure 9:**
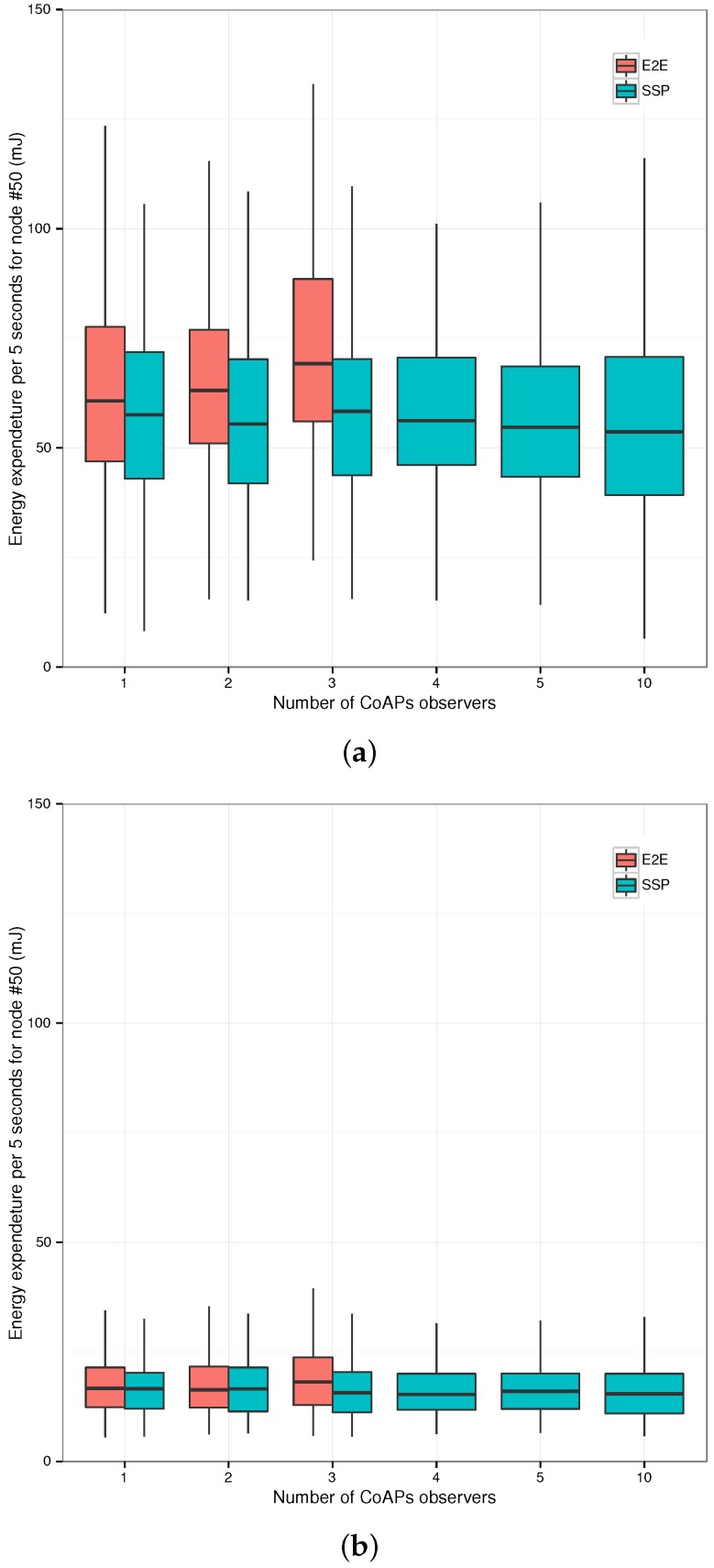
Total energy expenditure for Node #50 per five seconds interval for end-to-end (E2E) CoAPs observation versus CoAPS observation through the Smart Service Proxy (SSP). (**a**) One-second notification interval; (**b**) five-second notification interval.

When comparing the energy expenditure graphs for Node #50 in Figure 9, it becomes clear that aggregating CoAPs observation relationships leads to energy savings. The savings are proportional to the rate of notifications: they increase as the number of clients goes up and decrease as the notification interval becomes longer. Note that the sensor node between Node #50 and the border router experiences similar energy savings as every notification is received and retransmitted by this intermediary node. For the case of three CoAPs observers, the median energy expenditures differ by 10.8 mJ for the one-second interval and 2.5 mJ for the five-second interval.

For one CoAPs observer and the one second interval, there exists a small difference in energy expenditure between the end-to-end and the SSP case even though the notification rate is the same for both cases (i.e., one notification per second). This is primarily due to a difference in notification packet size, as the 6LoWPAN compression for SSP notifications is more effective than for E2E notifications. The compression is more effective because the IPv6 address of the SSP is part of the 6LoWPAN network, whereas the CoAPs client’s IPv6 address is part of a different network. As such, the prefix of the SSP’s IPv6 address can be elided (due to stateful 6LoWPAN compression), which leads to an eight-byte savings in packet size per notification.

**Figure 10:**
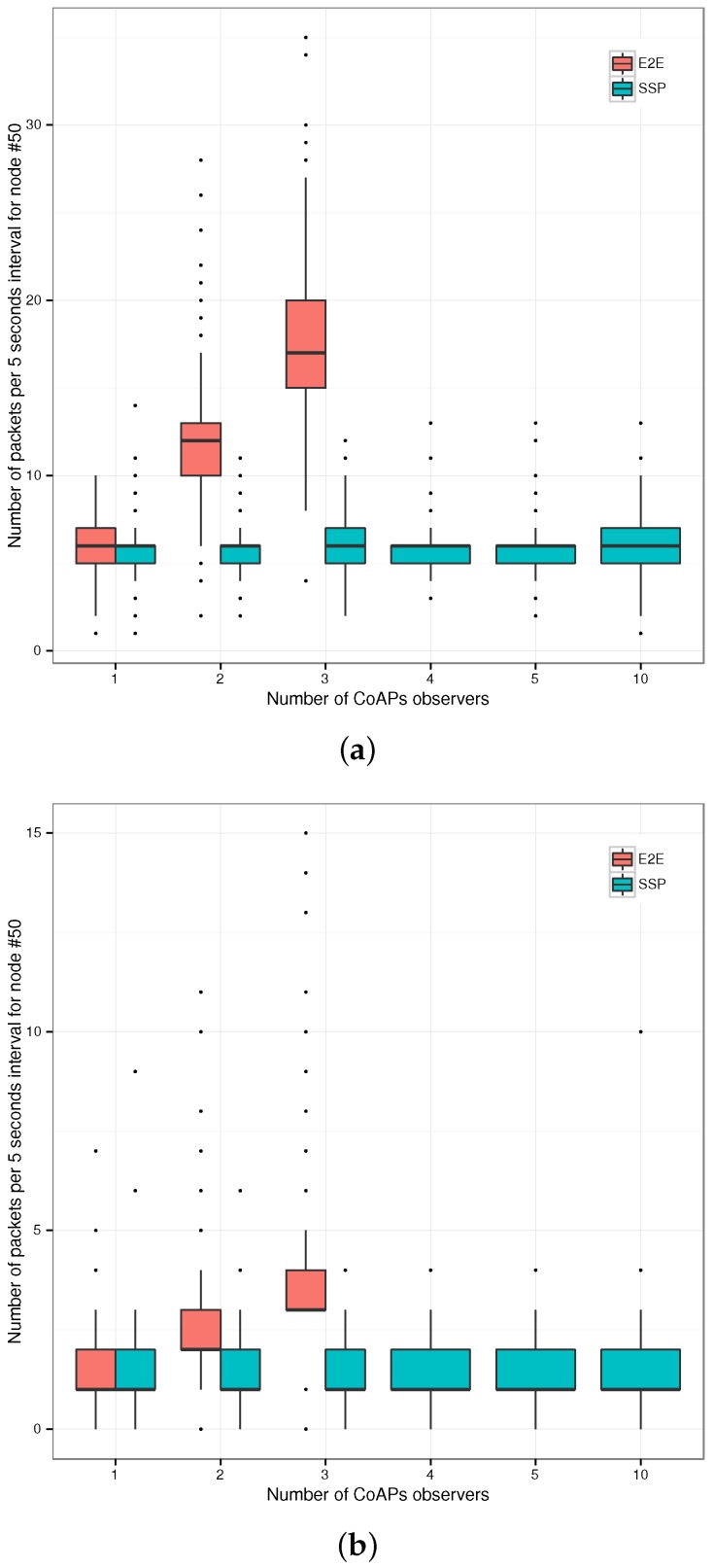
Number of exchanged packets for Node #50 per five-second interval for E2E CoAPs observation versus CoAPS observation through the SSP. (**a**) One-second notification interval; (**b**) five-second notification interval.

The graphs in Figure 10 clearly illustrate the difference in notification rate between the end-to-end and SSP experiments. Due to the aggregation of CoAPs observations at the SSP, there exists only one CoAPs observation between the SSP and the sensor node. This is illustrated in the constant notification rate for SSP as the number of CoAPs observers increases. For the end-to-end experiments, the notification rate rises linearly with the number of observers, as the sensor node sends notifications to each client separately. The slope of this linear relation is proportional to the notification frequency.

**Figure 11:**
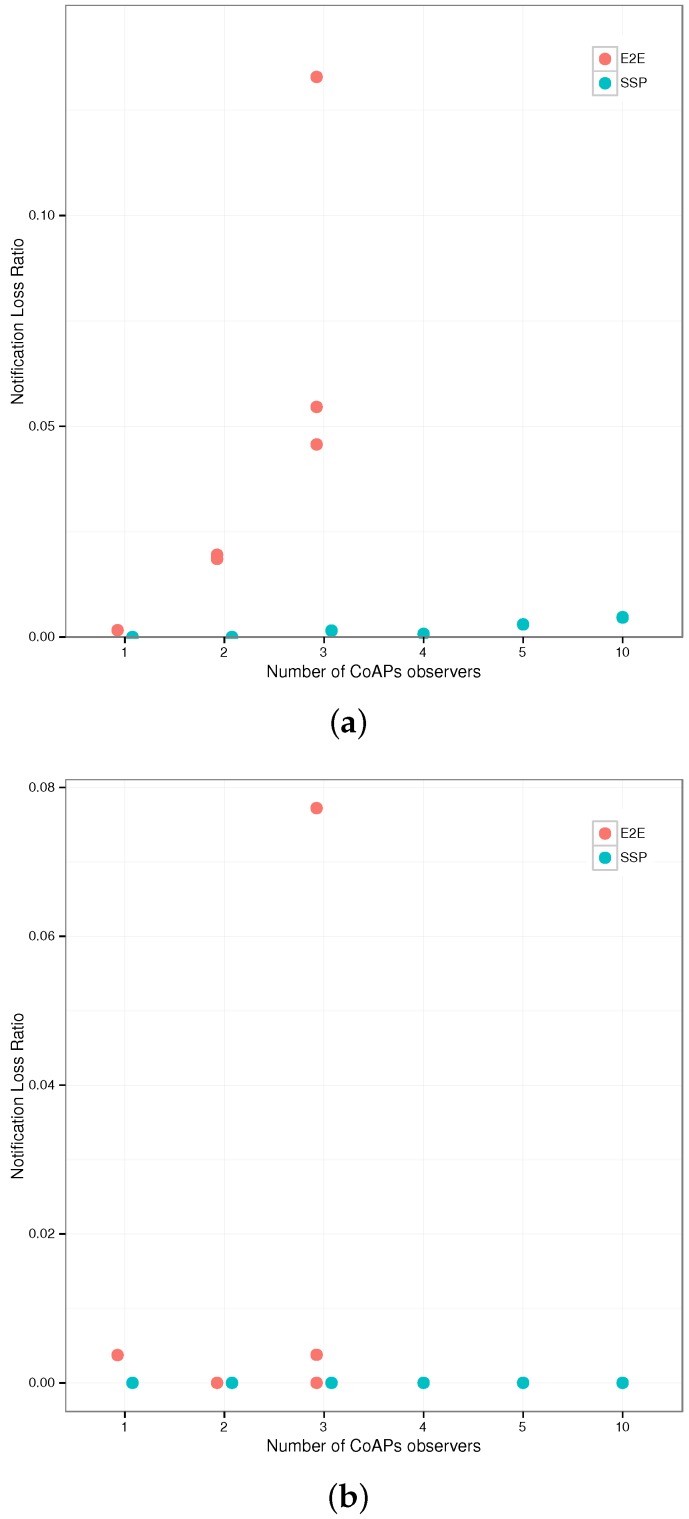
Notification loss ratios as measured at the CoAPs clients for E2E CoAPs observation versus CoAPS observation through the SSP. (**a**) One-second notification interval; (**b**) five-second notification interval.

Figure 11 plots the Notification Loss Ratios (NLR) for each of the eighteen experiments. For example for the E2E, one-second interval and one observer case, 1845 notifications were sent, three of which never arrived at the client. This leads to an NLR of 0.163%. Note that every vertical series of data contains as many points as there are observers; however, very similar and identical NLR’s overlap too much to distinguish them as separate points in the plot. The graphs for the one second interval show that the end-to-end case suffers from network congestion due to its higher notification rate. Furthermore, the observed loss is heavily dependent on the CoAPs client in the E2E experiments: i.e., the client that is last on the list of observers experiences the highest NLR (mostly apparent when there are three observers). Finally, the SSP sends every notification as a confirmable message. While in this setup, packet loss is mainly a problem in the constrained WSN, sending all notifications as CON messages can help to improve the NLR in situations where the client is a part of a lossy network.

To conclude, there are a number of limitations that are overcome by aggregating observations at the SSP:

1. Memory and processing constraints on the sensor node, which limit the number of simultaneously active DTLS sessions and active CoAP observation relationships.
2. Limited throughput in constrained (multi-hop) networks, which impacts the successful delivery of notifications and limits the rate of notifications.
3. Limited lifetime for battery-operated sensors: by reducing the load on constrained devices, the lifetime is lengthened.

Note that while only the results for Node #50 are shown, similar savings apply for other nodes. Also note that applying observation aggregation at the SSP delays the point at which the WSN reaches congestion, as the message rate in the WSN is reduced by the aggregation. Finally, note that this experiment is only possible because the SSP terminates the end-to-end security; indeed, should this not be the case, then the SSP would be unable to aggregate observation relationships, as all communications would be encrypted end-to-end.

## 7. Conclusions

In this work, we have presented the Secure Service Proxy: a CoAP(s) intermediary for use in resource-constrained RESTful environments. It has been designed to provide scalable end-to-end security for constrained devices and to extend constrained devices with additional functionality. The presented work follows a reverse proxy approach, where the SSP hosts virtual devices on behalf of resource-constrained devices. This approach enables the SSP to extend the virtual devices with security features that are hard to attain in constrained environments, such as authentication based on public key infrastructure (which, inherently, scales better than using PSKs), perfect forward secrecy and fine-grained authorization based on host identify and the nature of the request and resource. Additionally, the SSP extends virtual devices with a variety of different functions by means of an adapter chain system. Adapters are modular blocks of functionality that are hosted on the virtual device. Examples include caching, static resource and congestion control adapters. The SSP hosts a RESTful Web interface for managing virtual devices and adapter chains.

The SSP has been evaluated in two different setups. First, tests were performed in an LLN simulator to measure the effect of terminating end-to-end security on the SSP. The results of the simulator study demonstrate that session termination combined with long-term sessions in the constrained network leads to significant savings in network traffic, communication delay and processing and, consequently, leads to a longer battery life. The second study was run on a WSN testbed and quantified the impact of aggregating multiple observation relations with a constrained device over DTLS. The results confirm that the load on the constrained device and constrained network is independent of the number of observers. As a result, the packet rate and energy expenditure remain equal to those of the one observer case as the number of observers increases. Note that the session termination is a necessary condition for observation aggregation in case of DTLS-based security.

In conclusion, the presented Secure Service Proxy breaks end-to-end security in order to offer security primitives that are hard to attain on constrained systems while reducing the load on resource-constrained devices and networks. Additionally, the proxy provides extra application-layer features on behalf of constrained devices to services, which are built on top of these devices. Combined, the proxy facilitates the integration of constrained RESTful environments in services, thereby furthering the vision of an open, secure and scalable Web of Things.

## Figures and Tables

**Table 1:** The proxy offers a number of functionalities, called adapters, that are hosted on virtual devices. The list of adapters that were implemented at the time of this work are shown in this table.

Adapter	Functionality	Configuration Parameters
Access control	Restrict access to virtual devices depending on client identify, request method and URI.	ACL rules and default policy
Static resource	Host RESTful resources on virtual devices that can be read and modified.	Payload and content type
Cache	Cache and serve previous responses from virtual devices to clients.	Default cache entry lifetime
Congestion control	Enforce congestion control on clients querying virtual devices. Per-device and network-wide rules are implemented.	Per user CC limits
.well-known/core	Manipulate discovery responses from virtual devices to include functionality hosted by the proxy.	None
Proxy	Proxies requests for the virtual device to a CoAP(s) server (e.g., the constrained device). Also aggregates observation registrations.	CoAP(s) server endpoint
Mirror server	Proxies requests for a virtual device to a mirror server.	Mirror server endpoint and sleepy device anchor point
